# Right Ventricular–to–Pulmonary Artery Uncoupling Following Transcatheter Edge-to-Edge Repair in Ventricular Functional Mitral Regurgitation

**DOI:** 10.1016/j.jacasi.2026.03.040

**Published:** 2026-06-05

**Authors:** Daisuke Miyahara, Masaki Izumo, Shingo Kuwata, Taishi Okuno, Tetsu Tanaka, Yoshihiro J. Akashi, Masanori Yamamoto, Tetsuro Shimura, Atsushi Sugiura, Shunsuke Kubo, Mike Saji, Yuki Izumi, Yusuke Enta, Shinichi Shirai, Shingo Mizuno, Yusuke Watanabe, Makoto Amaki, Kazuhisa Kodama, Hisao Otsuki, Toru Naganuma, Hiroki Bota, Yohei Ohno, Masahiko Asami, Daisuke Hachinohe, Masahiro Yamawaki, Hiroshi Ueno, Gaku Nakazawa, Toshiaki Otsuka, Kentaro Hayashida, OCEAN-Mitral Investigators

**Affiliations:** a Department of Cardiology, St. Marianna University School of Medicine, Kawasaki, Japan; b Department of Cardiology, Toyohashi Heart Center, Toyohashi, Japan; c Department of Cardiology, Gifu Heart Center, Gifu, Japan; d Department of Cardiology, Nagoya Heart Center, Nagoya, Japan; e Department of Cardiology, Kurashiki Central Hospital, Kurashiki, Japan; f Department of Cardiology, Sakakibara Heart Institute, Tokyo, Japan; g Division of Cardiovascular Medicine, Department of Internal Medicine, Toho University Faculty of Medicine, Tokyo, Japan; h Department of Cardiology, Sendai Kosei Hospital, Sendai, Japan; i Division of Cardiology, Kokura Memorial Hospital, Kitakyushu, Japan; j Department of Cardiology, Shonan Kamakura General Hospital, Kanagawa, Japan; k Department of Cardiology, Teikyo University School of Medicine, Tokyo, Japan; l Department of Cardiology, National Cerebral and Cardiovascular Center, Suita, Japan; m Division of Cardiology, Saiseikai Kumamoto Hospital Cardiovascular Center, Kumamoto, Japan; n Department of Cardiology Tokyo Women’s Medical University, Tokyo, Japan; o Department of Cardiology, New Tokyo Hospital, Chiba, Japan; p Department of Cardiology, Sapporo Higashi Tokushukai Hospital, Sapporo, Japan; q Department of Cardiology, Tokai University School of Medicine, Isehara, Japan; r Division of Cardiology, Mitsui Memorial Hospital, Tokyo, Japan; s Department of Cardiology, Sapporo Heart Center, Sapporo Cardiovascular Clinic, Sapporo, Japan; t Department of Cardiology, Saiseikai Yokohama City Eastern Hospital, Kanagawa, Japan; u Second Department of Internal Medicine, Toyama University Hospital, Toyama, Japan; v Division of Cardiology, Department of Medicine, Kindai University Faculty of Medicine, Osaka, Japan; w Department of Hygiene and Public Health, Nippon Medical School, Tokyo, Japan; x Department of Cardiology, Keio University School of Medicine, Tokyo, Japan

**Keywords:** right ventricular function, RV-PA coupling, transcatheter edge-to-edge repair, ventricular functional mitral regurgitation

## Abstract

**Background:**

The prognostic value of right ventricular–pulmonary artery (RV-PA) coupling in patients with ventricular functional mitral regurgitation (FMR) undergoing mitral transcatheter edge-to-edge repair (M-TEER) is unclear.

**Objectives:**

The authors aimed to compare hospitalization and survival outcomes between patients with preserved and impaired RV-PA coupling.

**Methods:**

We analyzed 1,059 patients with ventricular FMR who underwent M-TEER. RV-PA coupling was assessed after M-TEER using the ratio of tricuspid annular plane systolic excursion (TAPSE) to systolic pulmonary artery pressure (SPAP). Impaired RV-PA coupling was defined as TAPSE/SPAP <0.36 mm/mm Hg. The primary outcome was a composite of cardiovascular death and hospitalization for heart failure.

**Results:**

Overall, 30.7% of patients exhibited impaired RV-PA coupling after M-TEER, with minimal improvement in TAPSE and SPAP. Patients with impaired RV-PA coupling at discharge had significantly lower event-free survival than those with preserved RV-PA (*P* = 0.004). Multivariate Cox regression analysis confirmed impaired RV-PA coupling as an independent predictor of the primary outcome (HR: 1.360; *P* = 0.029). The association between postprocedural RV-PA coupling and the primary outcome was more profound in patients with pulmonary hypertension (*P* = 0.047). Diabetes mellitus, impaired RV-PA coupling, lower left ventricular ejection fraction, smaller left ventricular size, and larger left atrial size before M-TEER were independent predictors of impaired RV-PA coupling.

**Conclusions:**

Impaired RV-PA coupling after M-TEER is independently associated with a higher risk of composite outcomes in patients with ventricular FMR, particularly those with pulmonary hypertension. Therefore, postprocedural assessment of RV-PA coupling may provide valuable prognostic insights and support risk stratification in this population.

Ventricular functional mitral regurgitation (FMR) is associated with poor prognosis in patients with heart failure (HF).^1^ Mitral transcatheter edge-to-edge repair (M-TEER) can alleviate symptoms due to FMR and reduce the risk of cardiovascular death and hospitalization for HF.^2^^,^^3^ In addition, right ventricular (RV) dysfunction has emerged as a prognostic marker in patients with ventricular FMR undergoing M-TEER^4^, ^5^, ^6^ because the RV myocardial performance is more sensitive to afterload changes compared with that of the left ventricle (LV).^7^ Therefore, systolic pulmonary artery pressure (SPAP), which reflects RV afterload, should be considered when evaluating RV systolic function.

Right ventricle–to–pulmonary artery (RV-PA) coupling, estimated noninvasively by echocardiography as the ratio of tricuspid annular plane systolic excursion (TAPSE) to SPAP (TAPSE/SPAP), is regarded as a surrogate for the RV length-force relationship and a prognostic marker in various populations with HF,^5^^,^^8^, ^9^, ^10^ including those with FMR.^11^ Furthermore, previous studies have shown that preprocedural RV-PA coupling and its change before and after M-TEER are prognostic indicators in patients with FMR.^12^, ^13^, ^14^ However, the prognostic utility of postprocedural RV-PA coupling in patients with ventricular FMR remains unclear. Therefore, we aimed to evaluate the prognostic significance and clinical predictors of impaired RV-PA coupling after M-TEER in patients with ventricular FMR, using data from a multicenter registry.

## Methods

### Study population

The OCEAN-Mitral (Optimized CathEter vAlvular iNtervention Mitral) registry is an ongoing, prospective, investigator-initiated, multicenter, observational registry of patients with mitral regurgitation (MR) undergoing M-TEER.^15^^,^^16^ This registry includes data from 21 Japanese centers and operates independently from industrial influence.

This registry enrolled 3,764 patients with primary and secondary MR who underwent M-TEER using the MitraClip G2 or G4 system (Abbott Vascular Inc) between April 2018 and June 2023. In the current study, only patients with echocardiographic evaluation at baseline and at discharge who underwent a successful M-TEER procedure for ventricular FMR (defined as residual MR ≤ moderate at discharge) were included. Data regarding baseline characteristics, procedural details, and clinical outcomes were systematically recorded. Each patient was evaluated by a multidisciplinary heart valve team comprising interventional cardiologists, cardiac surgeons, echocardiologists, and HF specialists. The indication for M-TEER in secondary MR was symptomatic despite optimal medical therapy and, if applicable, cardiac resynchronization therapy, in accordance with the Japanese guidelines.^17^

All patients provided informed consent. The study protocol was approved by the institutional review boards and conducted in accordance with the principles of the Declaration of Helsinki. This trial is registered with the University Hospital Medical Information Network (UMIN000023653).

### Transthoracic echocardiography

Comprehensive 2-dimensional and Doppler transthoracic echocardiography were performed before M-TEER and at discharge according to the American Society of Echocardiography (ASE) guidelines.^18^ All images were interpreted by experienced echocardiographers. MR severity was graded using qualitative and quantitative criteria and classified as none/trivial, mild, moderate, moderate to severe, or severe based on the ASE guidelines.^19^

The TAPSE was measured using M-mode echocardiography. SPAP was calculated using the following formula: 4 × (peak tricuspid regurgitation [TR] velocity)^2^+ mean right atrial pressure. The right atrial pressure was standardized to 8 mm Hg in accordance with the ASE guidelines to mitigate intercenter variability.^20^ Based on previous studies, impaired RV-PA coupling was defined as TAPSE/SPAP <0.36,^13^^,^^21^ lower TAPSE was defined as <17 mm, and pulmonary hypertension (PH) was defined as SPAP >40 mm Hg.^20^ In this study, the patients were divided into 2 groups according to their RV-PA coupling status. As a sensitivity analysis, we evaluated an alternative cutoff for impaired RV-PA coupling (TAPSE/SPAP <0.31), as previously reported.^22^

The etiology of MR was classified according to a previous study.^23^ MR was categorized as primary or secondary based on the echocardiographic findings. Patients with secondary MR were further classified into those with atrial and ventricular FMR. Atrial FMR was defined as: 1) absence of congenital or significant degenerative mitral valve abnormalities (prolapse, rheumatic change, or heavy calcification); 2) preserved LV systolic function (left ventricular ejection fraction [LVEF] >50%); and 3) absence of LV enlargement and regional wall motion abnormalities.

### Outcomes

Follow-up data were collected from the medical records and telephone interviews. The primary outcome of the study was the time to the first occurrence of a composite endpoint, defined as cardiovascular death or hospitalization for HF. Secondary outcomes included the individual components of the primary endpoint and all-cause mortality.

### Statistical analysis

Patients were stratified into 2 groups based on postprocedural RV-PA coupling status: impaired (TAPSE/SPAP <0.36) and normal (≥0.36). Continuous variables were expressed as mean ± SD or median (Q1-Q3). Categorical variables were expressed as n(%). Student’s *t*-test was used to analyze continuous variables with normal distributions, and the Mann-Whitney *U* test was used to analyze continuous variables with non-normal distributions. Categorical variables were compared using Fisher’s exact or chi-square tests, as appropriate. The cumulative probability of event-free survival was estimated using the Kaplan-Meier method and compared between groups using a log-rank test. The univariable and multivariable Cox proportional hazard models were used to calculate HRs and 95% CIs for the primary outcome. In addition, HRs were reported after adjustments for other prognostic variables including age, sex, clinical frailty score, body mass index (BMI), NYHA functional classification III-IV, diabetes mellitus (DM), atrial fibrillation/flutter (AF/AFL), estimated glomerular filtration rate (eGFR) at baseline, LVEF and systolic blood pressure after M-TEER, and residual MR (≥ moderate), transmitral gradient (TMG) (≥4 mm Hg), and significant TR (≥ moderate to severe) after M-TEER. The proportional hazards assumption was assessed using Schoenfeld residuals. For variables violating this assumption, stratified Cox proportional hazards models were applied. Multicollinearity was evaluated using variance inflation factors (VIFs). Potential nonlinear associations were explored using restricted cubic splines. Given the substantial proportion of missing TAPSE and SPAP data, we performed a sensitivity analysis using inverse probability weighting (IPW) to mitigate potential bias due to missingness. The probability of missing TAPSE/SPAP data was estimated using a multivariable logistic regression model incorporating the same prespecified clinically relevant covariates as in the primary multivariable analyses. Stabilized inverse probability weights were calculated and applied to Cox proportional hazards models. Covariate balance before and after weighting was evaluated using standardized mean differences (SMDs). The effective sample size (ESS) was calculated as (Σw)^2^/Σw^2^ to assess the stability of the weighted analysis. This approach assumes that data are missing at random conditional on observed covariates included in the missingness model.

For the multivariable Cox regression analysis, covariates were prespecified a priori based on their clinical relevance and established associations with impaired RV-PA coupling after M-TEER. The adjusted model included age, sex, BMI, Clinical Frailty Scale, AF/AFL, hypertension, DM, hospitalization for HF, prior cardiac surgery, cardiac resynchronization therapy, hemoglobin, eGFR, B-type natriuretic peptide (BNP), systolic blood pressure, LVEF, LV end-diastolic dimension, left atrial (LA) dimension, effective regurgitant orifice area, significant TR, and impaired RV-PA coupling before M-TEER. Residual MR (moderate) and TMG (≥4 mm Hg) after M-TEER were treated as ordinal variables and entered as linear terms (per 1-class increase) in the multivariable Cox models. To assess collinearity, VIFs were calculated for all covariates; all VIFs were <5.0, indicating minimal collinearity.

To explore potential effect modification, subgroup analyses were conducted across clinically relevant categories, including sex, age, BMI, AF/AFL, NYHA functional class (I-II vs III-IV), PH, and baseline RV-PA coupling. Multiplicative interaction terms were formally tested by including cross-product terms in the Cox proportional hazards models. All data analyses were performed using the R statistical software (version 4.2.2; R Foundation for Statistical Computing).

## Results

### Study population

**Figure 1:**
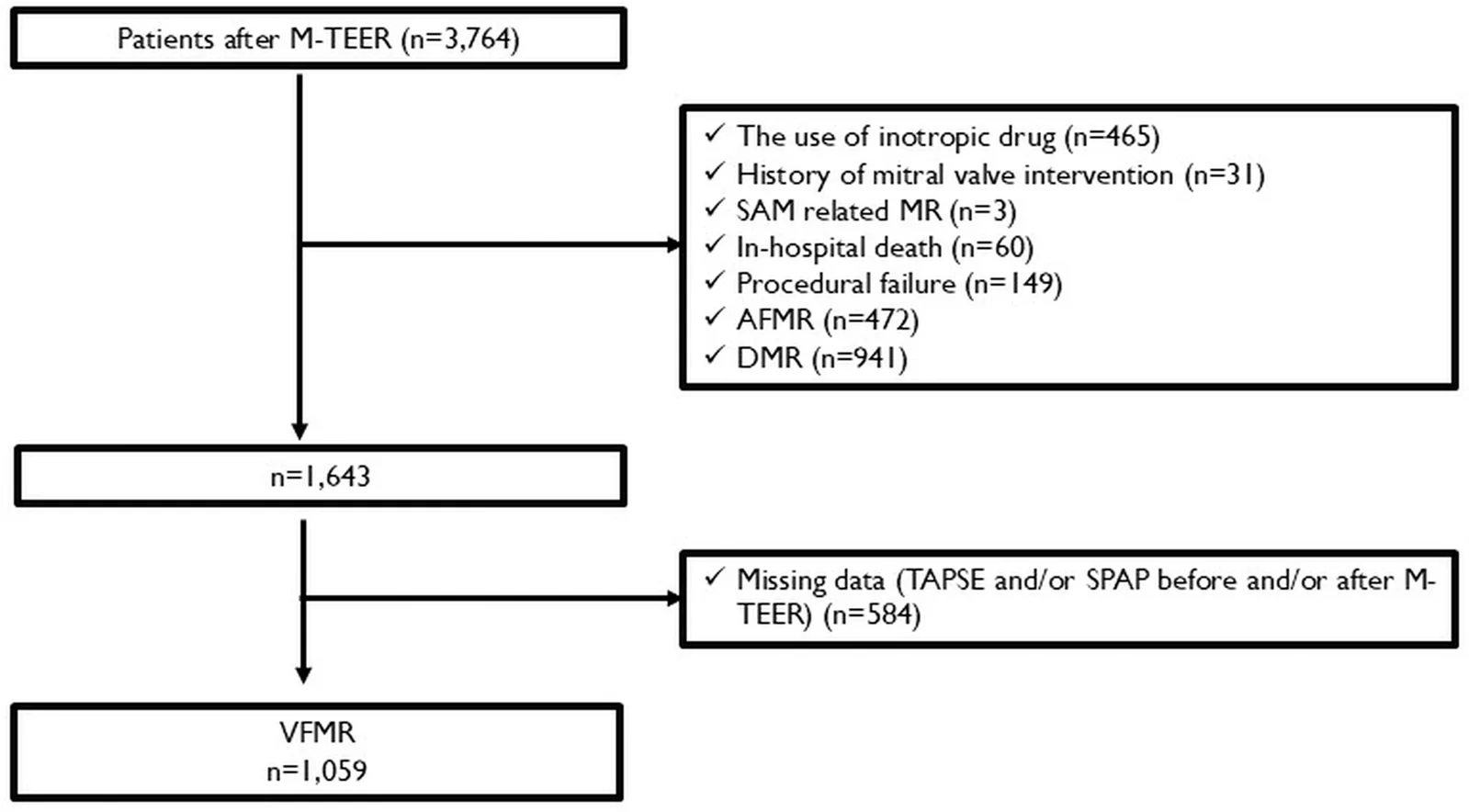
Study Flow Diagram This figure illustrates the selection of the study population from the OCEAN-Mitral registry. Among 3,764 patients undergoing M-TEER, those with VFMR and complete echocardiographic data at baseline and discharge were included (n = 1,059). AFMR = atrial functional mitral regurgitation; DMR = degenerative mitral regurgitation; M-TEER = mitral transcatheter edge-to-edge repair; MR = mitral regurgitation; SAM = systolic anterior motion; SPAP = systolic pulmonary artery pressure; TAPSE = tricuspid annular plane systolic excursion; VFMR = ventricular functional mitral regurgitation.

Of the 3,764 patients who underwent M-TEER, with data in the OCEAN-Mitral registry, between April 2018 and June 2023, 1,059 with ventricular FMR and complete echocardiographic data were included in the present study. Patients were excluded because of nonventricular FMR etiologies, prior mitral valve interventions, in-hospital death, procedural failure, use of inotropes before the procedure, or missing echocardiographic parameters such as TAPSE or SPAP (Figure 1). Supplemental Table 1 presents the comparison of patient characteristics between the included and excluded patients with ventricular FMR and those with missing data. Although baseline characteristics differed between patients with and without missing TAPSE/SPAP data, these differences were largely explained by observed covariates included in the IPW model.

No significant differences were observed in the composite endpoint between the included and excluded patients in the sensitivity analysis (*P* = 0.524) (Supplemental Figure 1). Among 1,643 eligible patients, 1,366 with complete data for covariates included in the missingness model were analyzed in the IPW sensitivity analysis. Although baseline characteristics differed between patients with and without missing TAPSE/SPAP data, these differences were substantially attenuated after weighting. Most SMDs were <0.1 following weighting (Supplemental Table 2), indicating adequate covariate balance. The ESS after weighting was 1,331, close to the analytic sample size, suggesting that the results were not driven by extreme weights.

### Baseline characteristics

Impaired RV-PA coupling after M-TEER was observed in 325 patients (30.7%) and their baseline characteristics are summarized in Table 1. Patients with impaired RV-PA coupling were older, had a lower proportion of men, more frequently presented with NYHA functional class III-IV symptoms, and had higher rates of previous cardiac surgery and AF/AFL compared with those with preserved RV-PA coupling. In addition, patients with impaired RV-PA coupling had higher Society of Thoracic Surgeons and EuroSCORE II scores, lower hemoglobin and eGFR, and higher BNP/N-terminal pro–B-type natriuretic peptide (NT-proBNP) levels compared with those with preserved RV-PA coupling. No significant differences were observed in the prescription rates of cardioprotective drugs or diuretics between the 2 groups.

**Table 1:** Baseline Characteristics

	Impaired RV-PA Coupling (n = 325)	Preserved RV-PA Coupling (n = 734)	*P* Value
Male	192 (59.1)	495 (67.4)	0.010
Age, y	79.0 (72.0-83.0)	77.0 (70.0-82.0)	0.035
BMI, kg/m^2^	21.4 (19.1-22.8)	21.4 (19.0-23.6)	0.371
BSA, m^2^	1.52 (1.38-1.66)	1.56 (1.43-1.68)	0.007
Hypertension	209 (64.3)	462 (62.9)	0.722
Diabetes mellitus	121 (37.2)	238 (32.4)	0.146
CKD	297 (91.4)	645 (87.9)	0.115
Dialysis	31 (9.5)	55 (7.5)	0.316
Dyslipidemia	172 (52.9)	411 (56.0)	0.390
Coronary artery disease	148 (45.5)	309 (42.1)	0.329
Previous PCI	115 (35.4)	260 (35.4)	1.000
Previous CABG	42 (12.9)	33 (4.5)	<0.001
COPD	30 (9.2)	61 (8.3)	0.708
AF/AFL	217 (66.8)	425 (57.9)	0.008
Prior hospitalization for heart failure	276 (87.6)	629 (88.1)	0.911
Previous cardiac surgery	54 (16.6)	63 (8.6)	<0.001
CRT	55 (16.9)	111 (15.1)	0.515
NYHA functional class Ⅲ-Ⅳ	218 (67.1)	416 (56.7)	0.002
Clinical frailty scale	3.0 (3.0-4.0)	3.0 (3.0-4.0)	0.107
β-blocker	280 (86.2)	636 (86.6)	0.905
ACE inhibitor/ARB	186 (57.2)	441 (60.1)	0.422
ARNI	30 (9.2)	82 (11.2)	0.402
MRA	186 (57.2)	446 (60.8)	0.311
Diuretics	289 (88.9)	645 (87.9)	0.701
SGLT2 inhibitor	79 (24.3)	178 (24.3)	1.000
EuroSCORE Ⅱ	6.42 (3.99-11.42)	5.11 (3.28-8.20)	<0.001
STS	9.70 (6.04-15.40)	8.31 (5.02-12.72)	<0.001
Hb, g/dL	11.8 (10.6-13.0)	12.1 (10.9-13.4)	0.018
Cre, mg/dL	1.45 (1.07-2.12)	1.31 (1.02-1.87)	0.016
eGFR, mL/min/1.73 m^2^	33.8 (22.4-45.4)	37.1 (25.2-50.5)	0.003
BNP, pg/mL	573.9 (288.6-1066.1)	376.6 (202.4-639.0)	<0.001
NT-pro BNP, pg/mL	4,674.0 (2,190.9-9563.5)	2,787.0 (1,476.8-5,409.3)	<0.001
Number of clips			0.201
1	225 (69.2)	543 (74.0)	
2	95 (29.2)	185 (25.2)	
3	5 (1.5)	6 (0.8)	

Values are median (Q1-Q3) or n (%).

ACE = angiotensin-converting enzyme; AF = atrial fibrillation; AFL = atrial flutter; ARB = angiotensin Ⅱ receptor blocker; ARNI = angiotensin receptor neprilysin inhibitor; BMI = body mass index; BNP = B-type natriuretic peptide; BSA = body surface area; CABG = coronary artery bypass grafting; CKD = chronic kidney disease; COPD = chronic obstructive pulmonary disease; Cre = creatinine; CRT = cardiac resynchronization therapy; eGFR = estimated glomerular filtration rate; EuroScore = European system of cardiac operative risk evaluation; Hb = hemoglobin; MRA = mineralocorticoid receptor antagonist; NT-proBNP = N-terminal pro–B-type natriuretic peptide; PCI = percutaneous coronary artery intervention; RV-PA = right ventricular–pulmonary artery; SGLT = sodium-glucose cotransporter 2; STS = Society of Thoracic Surgeons risk score.

### Echocardiographic characteristics before and after M-TEER

The echocardiographic findings before and after M-TEER are presented in Tables 2 and 3. Before M-TEER, the impaired RV-PA coupling group had a larger LA size and RV mid-diameter, lower stroke volume, TAPSE, fractional area change, s’ of the tricuspid annulus, and TAPSE/SPAP ratio, and higher SPAP, E wave, and E/e’ and more severe TR compared with those with preserved RV-PA coupling. The LV volume, LVEF, and MR severity were not significantly different between the 2 groups before M-TEER.

**Table 2:** Echocardiographic Characteristics Before M-TEER

	Impaired RV-PA Coupling (n = 325)	Preserved RV-PA Coupling (n = 734)	*P* Value
Systolic blood pressure, mm Hg	102.0 (94.0-114.0)	105.0 (94.0-118.0)	0.213
Heart rate, beats/min	74.0 (66.0-84.0)	71.0 (62.0-80.0)	0.001
LVDd, mm	60.0 (54.0-67.0)	62.0 (56.0-68.0)	0.023
LVDs, mm	50.0 (42.0-58.0)	52.0 (45.0-59.0)	0.008
LVEDV, mL	167.0 (119.0-206.6)	169.0 (129.0-213.0)	0,159
LVESV, mL	108.0 (71.0-151.0)	112.0 (78.0-152.0)	0.265
LVEF, %	33.3 (27.6-41.4)	32.9 (26.9-40.4)	0.357
LAD, mm	51.0 (46.0-56.0)	48.0 (43.0-54.0)	<0.001
LAV index, mL/m^2^	81.3 (64.2-108.8)	68.4 (54.7-92.3)	<0.001
Stroke volume, mL	41.8 (33.1-53.8)	46.8 (36.6-58.9)	<0.001
SPAP, mmHg	43.0 (36.0-54.4)	36.0 (30.0-46.0)	<0.001
TAPSE, mm	13.2 (11.0-16.0)	16.3 (13.5-19.5)	<0.001
TAPSE/SPAP	0.29 (0.23-0.39)	0.44 (0.33-0.57)	<0.001
RV s’, cm/s	8.0 (6.5-9.7)	9.0 (7.3-11.0)	<0.001
FAC, %	30.9 (25.0-38.0)	36.0 (28.0-44.0)	<0.001
RVDd-mid, mm	31.0 (26.9-35.8)	29.0 (25.0-34.0)	0.001
MR grade			0.122
≤ moderate	37 (11.3)	125 (17.1)	
Moderate to severe	117 (36.0)	231 (31.5)	
Severe	171 (52.6)	378 (51.5)	
MR ERO, cm^2^	0.31 (0.24-0.40)	0.30 (0.22-0.40)	0.152
TR grade			<0.001
Moderate	108 (33.2)	175 (23.9)	
Moderate to severe	30 (9.2)	44 (6.0)	
Severe	5 (1.5)	5 (0.7)	
E/e’	18.3 (13.4-23.4)	16.1 (12.2-20.9)	0.001
E, cm/s	99.0 (78.0-120.3)	90.0 (70.2-109.0)	<0.001

Values are median (Q1-Q3) or n (%).

ERO = effective regurgitant orifice area; FAC = fractional area change; LAD = left atrial dimension; LAV = left atrial volume; LVDd = left ventricular diastolic dimension; LVDs = left ventricular systolic dimension; LVEDV = left ventricular end-diastolic volume; LVEF = left ventricular ejection fraction; LVESV = left ventricular end-systolic volume; MR = mitral regurgitation; M-TEER = mitral transcatheter edge-to-edge repair; RV = right ventricular; RVDd = right ventricular diastolic dimension; SPAP = systolic pulmonary artery pressure; TAPSE = tricuspid annular plane systolic excursion; TR = tricuspid regurgitation; other abbreviation as in Table 1.

**Table 3:** Echocardiographic Characteristics After M-TEER

	Impaired RV-PA Coupling (n = 325)	Preserved RV-PA Coupling (n = 734)	*P* Value
Systolic blood pressure, mm Hg	104.0 (95.0-115.0)	102.0 (92.0-115.0)	0.174
Heart rate, beats/min	70.0 (63.0-79.0)	68.0 (60.0-77.0)	0.004
LVDd, mm	59.0 (51.0-65.2)	60.0 (54.0-66.0)	0.008
LVDs, mm	50.0 (41.0-58.0)	52.0 (44.0-58.8)	0.019
LVEDV, mL	152.5 (109.3-209.8)	159.0 (122.0-206.0)	0.204
LVESV, mL	106.0 (68.5-153.0)	110.0 (76.0-147.0)	0.324
LVEF, %	30.6 (24.7-38.5)	31.4 (25.5-39.4)	0.353
LAD, mm	49.0 (44.8-54.0)	47.0 (42.0-52.0)	<0.001
LAV index, mL/m^2^	77.2 (59.9-103.4)	66.0 (51.4-87.4)	<0.001
Stroke volume, mL	43.3 (34.3-53.5)	48.8 (39.2-61.7)	<0.001
SPAP, mm Hg	42.0 (36.0-50.0)	33.0 (29.0-38.0)	<0.001
ΔSPAP, mm Hg	−2.0 ( −9.0 to 4.0)	−3.0 ( −11.0 to 2.0)	0.002
SPAP > 40 mm Hg	182 (56.0)	132 (18.0)	<0.001
TAPSE, mm	11.9 (9.9-14.0)	17.0 (14.6-20.0)	<0.001
ΔTAPSE	−1.0 ( −4.0 to 0.8)	0.85 ( −1.8 to 3.3)	<0.001
TAPSE/SPAP	0.29 (0.24-0.33)	0.50 (0.43-0.60)	
ΔTAPSE/SPAP	−0.02 (0.10-0.04)	0.07 ( −0.03 to 0.18)	<0.001
MR grade			0.553
moderate	68 (20.9)	133 (18.1)	
TMG, mmHg	2.80 (2.00-3.70)	2.30 (1.80-3.30)	0.005
TR, grade			<0.001
Moderate	85 (26.2)	117 (16.0)	
Moderate to severe	27 (8.3)	30 (4.1)	
Severe	2 (0.6)	5 (0.7)	
E/e’	25.0 (19.7-32.6)	22.9 (18.2-29.7)	0.011
E, cm/s	112.5 (84.5-137.0)	97.0 (74.0-118.0)	<0.001

Values are median (Q1-Q3) or n (%).

TMG = transmitral gradient; other abbreviations as in Table 2.

After M-TEER, patients with impaired RV-PA coupling had significantly lower stroke volume and TAPSE, higher SPAP and transmitral mean pressure gradient, and worse TR severity compared with those with preserved RV-PA coupling. No significant differences were observed in LVEF and residual MR between the 2 groups.

**Figure 2:**
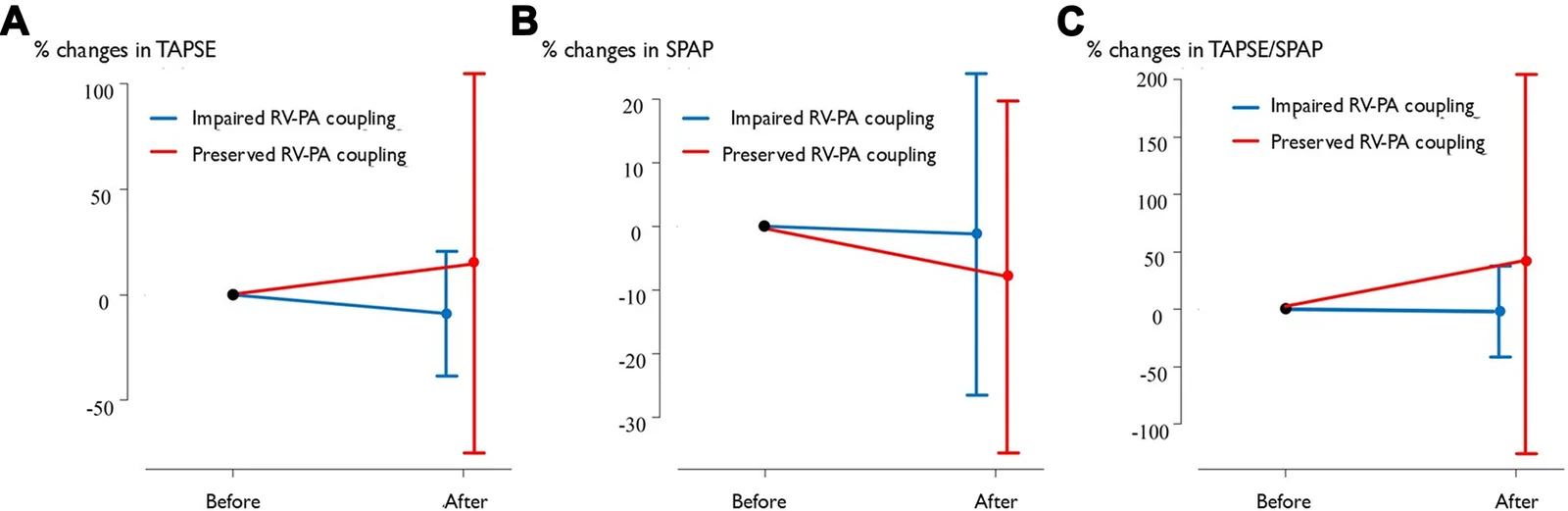
RV-PA Coupling Changes After M-TEER This figure shows longitudinal changes in (A) TAPSE, (B) SPAP, and (C) TAPSE/SPAP before and after M-TEER. RV-PA = right ventricular–pulmonary artery; other abbreviations as in Figure 1.

The changes in TAPSE, SPAP, and TAPSE/SPAP before and after M-TEER are shown in Figure 2. The impaired RV-PA coupling group showed significantly less improvement in TAPSE and SPAP after M-TEER than did the impaired RV-PA coupling group.

### Outcomes according to RV-PA coupling

**Figure 3:**
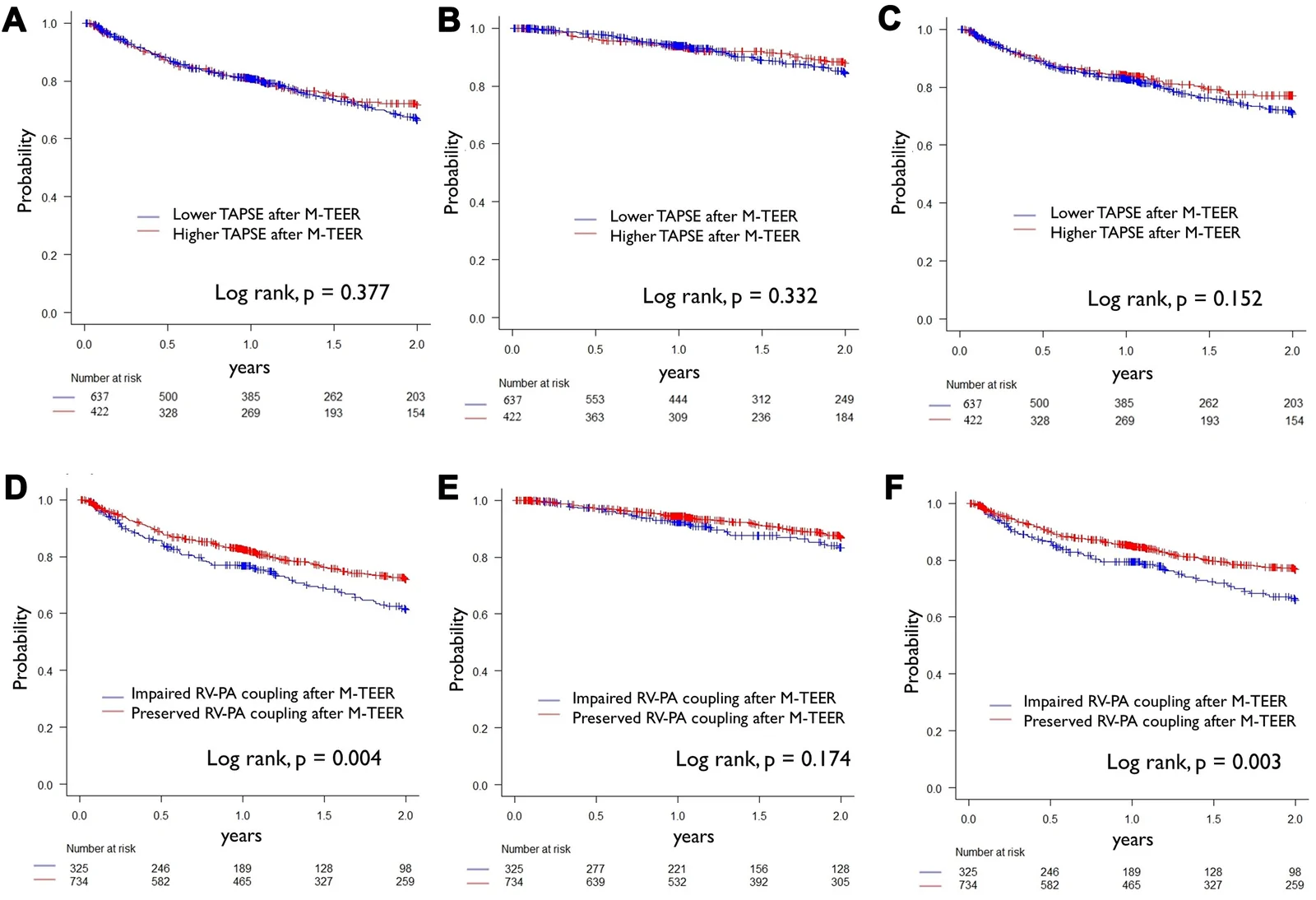
Outcomes by TAPSE and RV-PA Coupling Kaplan-Meier curves compare clinical outcomes stratified by TAPSE and RV-PA coupling after M-TEER. (A) Composite endpoints (cardiovascular death and hospitalization for heart failure) (*P* = 0.377), (B) cardiovascular death (*P* = 0.332) and (C) hospitalization for heart failure (*P* = 0.152), stratified by TAPSE after M-TEER. (D) Composite endpoints (cardiovascular death and hospitalization for heart failure) (*P* = 0.004), (E) cardiovascular death (*P* = 0.174), and (F) hospitalization for heart failure (*P* = 0.003), stratified by RV-PA coupling after M-TEER. Abbreviations as in Figures 1 and 2.

During a median follow-up period of 414 days (IQR: 222-731 days), the composite endpoint occurred in 260 patients: cardiovascular death and hospitalization for HF in 107 and 218 patients, respectively. No significant difference was observed in the composite endpoint, cardiovascular death and hospitalization for HF between the patients with lower and higher TAPSE after M-TEER (log-rank test, *P* = 0.377, 0.332, and 0.152) (Figures 3A to 3C). In contrast, Kaplan-Meier analysis demonstrated that patients with impaired RV-PA coupling after M-TEER had a significantly lower event-free survival rate than those with preserved RV-PA coupling (log-rank test, *P* = 0.004) (Figure 3D). Similarly, hospitalization for HF was more frequent in patients with impaired RV-PA coupling after M-TEER compared with those with preserved RV-PA coupling after M-TEER (log-rank test, *P* = 0.003) (Figure 3F), whereas no difference was observed in cardiovascular mortality between the 2 groups (Figure 3E). In this sensitivity analysis using the alternative RV-PA coupling threshold (TAPSE/SPAP <0.31), Kaplan-Meier curves showed consistent risk separation compared with the primary prespecified cutoff (log-rank test, *P* < 0.001) (Supplemental Figure 2).

**Figure 4:**
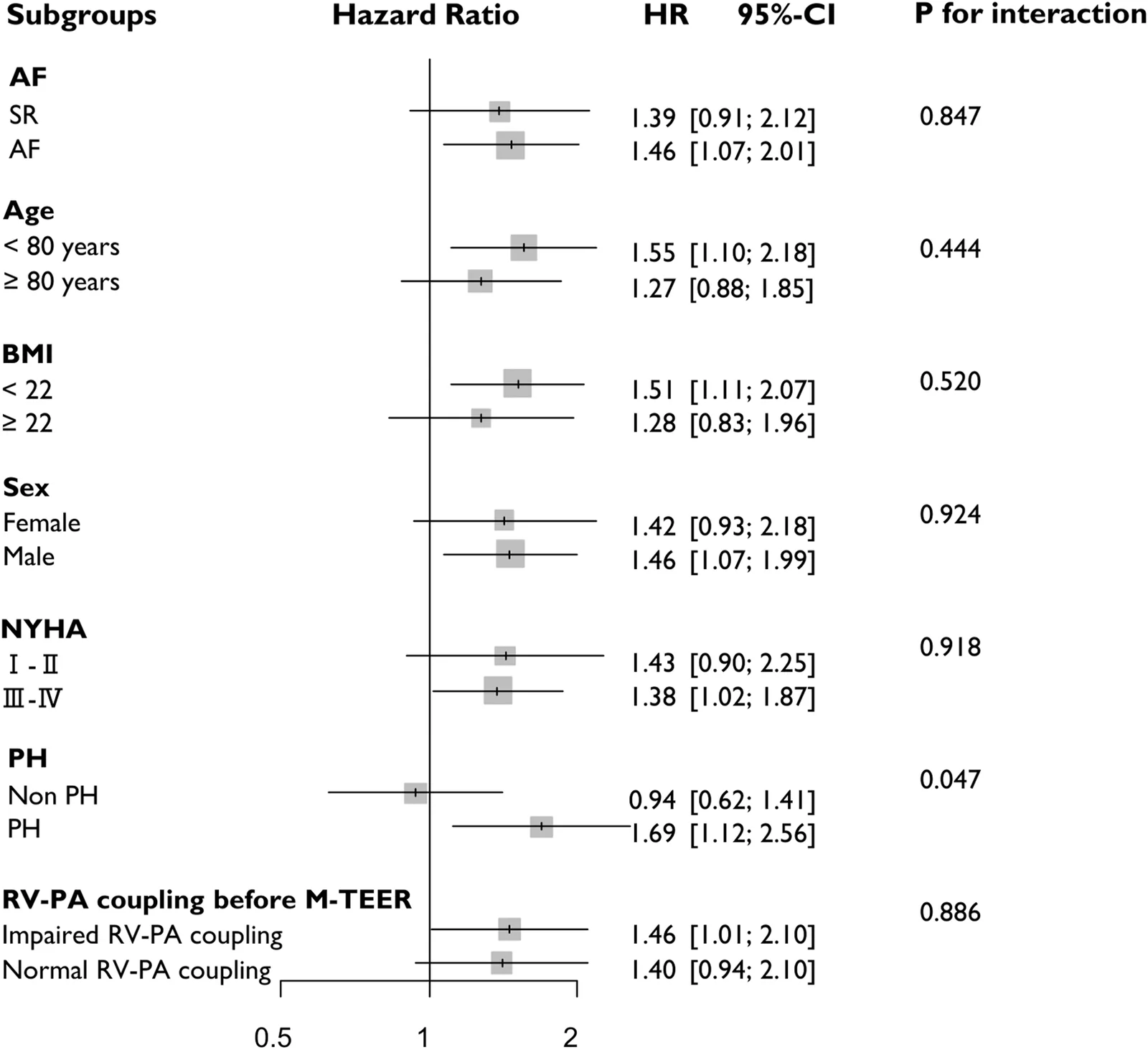
Subgroup Analysis for Composite Endpoint Forest plot displaying HRs for the composite endpoint according to RV-PA coupling status across clinically relevant subgroups. Impaired RV-PA coupling was consistently associated with worse outcomes, with a stronger effect observed in patients with PH. AF = atrial fibrillation; BMI = body mass index; PH = pulmonary hypertension; SR = sinus rhythm; other abbreviations as sin Figures 1 and 2.

Kaplan-Meier analysis based on RV-PA coupling before M-TEER did not demonstrate effective prognostic stratification (Supplemental Figures 3A to 3C). Therefore, RV-PA coupling after M-TEER was analyzed and stratified by the presence of RV-PA coupling before M-TEER. The results showed that RV-PA coupling after M-TEER was effective for prognostic stratification, regardless of RV-PA coupling before M-TEER (*P* for interaction = 0.886) (Figure 4, Supplemental Figure 4).

### Trajectory of RV-PA coupling before and after M-TEER

**Figure 5:**
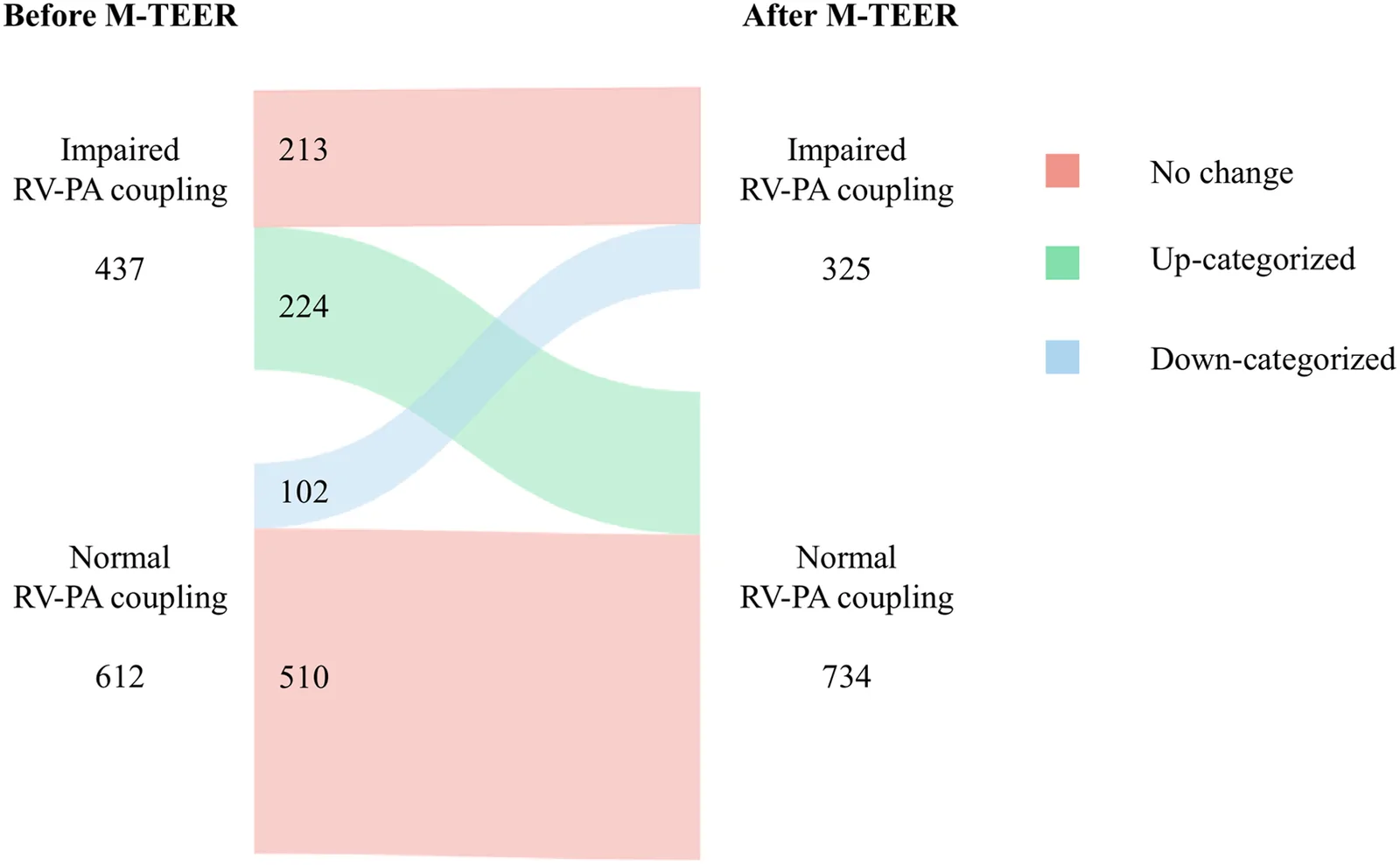
RV-PA Coupling Trajectories This figure depicts transitions in RV-PA coupling status before and after M-TEER. Among patients with impaired RV-PA coupling before M-TEER, 224 (51.3%) improved to normal coupling after the procedure. Conversely, 102 (16.7%) patients with normal baseline coupling deteriorated to impaired status after M-TEER. Abbreviations as in Figures 1 and 2.

The trajectory of RV-PA coupling before and after M-TEER is shown in Figure 5. Among patients with impaired RV-PA coupling before M-TEER, 224 (51.3%) improved to normal coupling after the procedure. Conversely, 102 (16.7%) patients with normal baseline coupling deteriorated to impaired status after M-TEER. We analyzed baseline characteristics and echocardiographic parameters across the 4 trajectory groups (Supplemental Tables 3 to 5). The group with improved RV-PA coupling demonstrated a significant reduction in SPAP after M-TEER. In contrast, the group with deterioration showed a significant decline in TAPSE and a higher prevalence of significant TR (≥ moderate) after M-TEER.

### Univariable and multivariable Cox regression analysis

The proportional hazards assumption was satisfied for the overall model (global test *P* = 0.650). Although TMG ≥4 mm Hg after M-TEER demonstrated evidence of nonproportionality (*P* = 0.016), stratified Cox modeling yielded consistent HRs for impaired RV-PA coupling and PH. All VIFs were <5, indicating no significant multicollinearity (Supplemental Table 6). Restricted cubic spline analysis showed no significant evidence of nonlinearity, supporting a linear association between postprocedural RV-PA coupling and the composite outcome. The results of the IPW-adjusted analysis were consistent with those of the primary complete-case analysis (HR: 1.416; 95% CI: 1.062-1.887 and HR: 1.348; 95% CI: 1.021-1.779; *P* = 0.018 and 0.035) (Table 4). The RV-PA coupling status effectively stratified prognosis in patients with PH after M-TEER (*P* for interaction = 0.047) (Figure 4, Central Illustration).

**Table 4:** Univariable and Multivariable Cox Regression Analyses for Predicting the Primary Outcomes

	Univariable Model		Multivariable Model^a^		IPTW^b^	
	HR (95% CI)	*P* Value	HR (95% CI)	*P* Value	HR (95% CI)	*P* Value
Impaired RV-PA coupling after M-TEER	1.440 (1.120-1.851)	0.005	1.365 (1.031-1.806)	0.030	1.348 (1.021-1.779)	0.035
Lower TAPSE after M-TEER	1.131 (0.880-1.453)	0.337	1.068 (0.801-1.423)	0.655	1.064 (0.802-1.412)	0.666
PH after M-TEER	1.595 (1.244-2.047)	<0.001	1.433 (1.069-1.920)	0.016	1.416 (1.062-1.887)	0.018

IPTW = inverse probability of treatment weighting; PH = pulmonary hypertension; other abbreviations as in Tables 1 and 2.

a Adjusted for age, sex, clinical frailty score, body mass index, NYHA functional classification, diabetes mellitus, atrial fibrillation/flutter, estimated glomerular filtration rate at baseline, and left ventricular ejection fraction, systolic blood pressure, residual mitral regurgitation (moderate), and significant tricuspid regurgitation (≥ moderate to severe) after M-TEER. The model was stratified by transmitral gradient (≥4 mm Hg) after M-TEER to account for nonproportionality.

b Adjustment was performed using the IPTW approach and Cox multivariable adjustment.

**Central Illustration:**
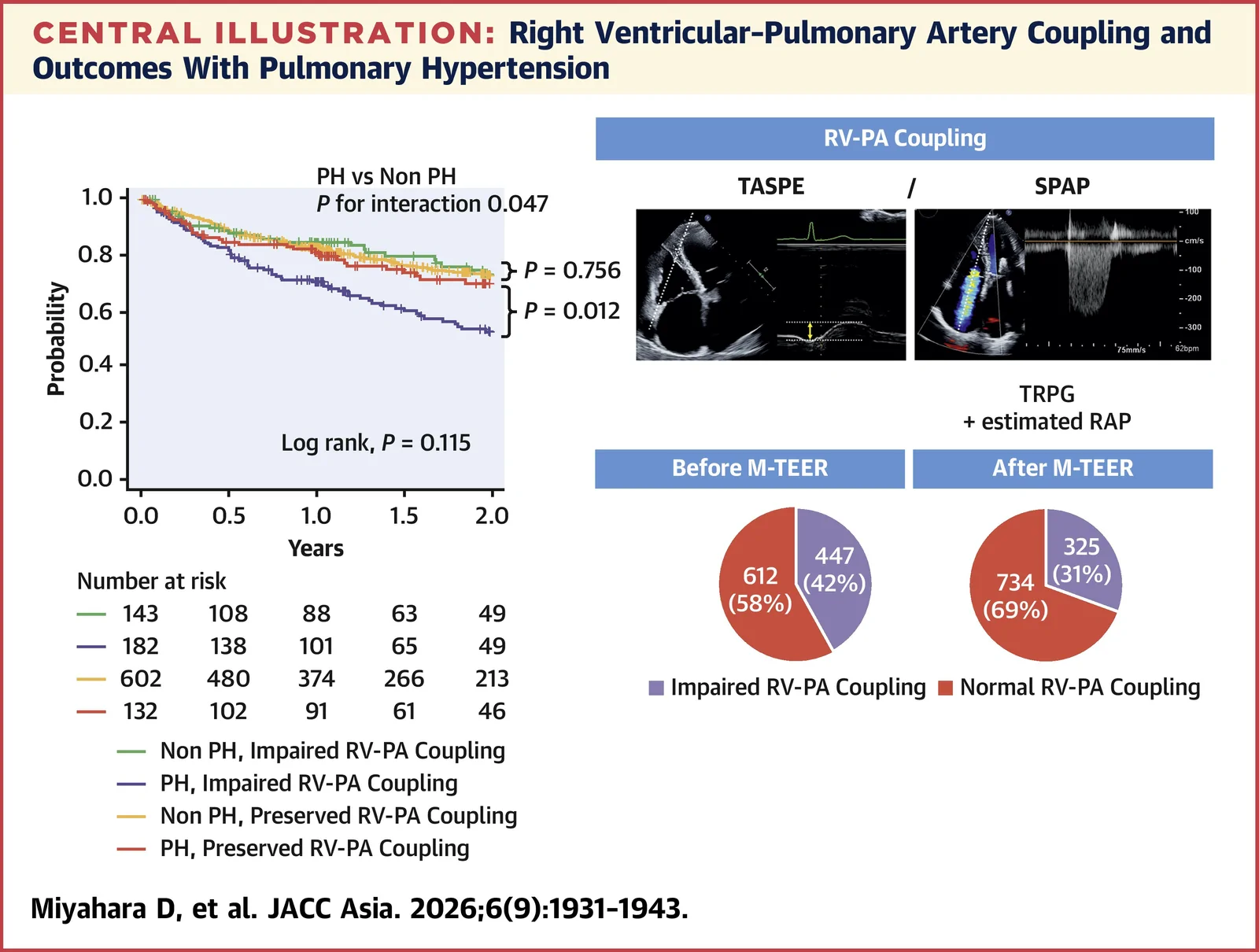
Right Ventricular–Pulmonary Artery Coupling and Outcomes With Pulmonary Hypertension Kaplan-Meier curves illustrate the impact of RV-PA coupling stratified by PH status after M-TEER. Patients with impaired RV-PA coupling and concomitant PH demonstrated the highest risk of cardiovascular death or heart failure hospitalization. M-TEER = mitral transcatheter edge-to-edge repair; PH = pulmonary hypertension; RAP = right atrial pressure; RV-PA = right ventricular–pulmonary artery; SPAP = systolic pulmonary artery pressure; TAPSE = tricuspid annular plane systolic excursion; TRPG = tricuspid regurgitation pressure gradient.

### Predictors of impaired RV-PA coupling after M-TEER

Univariable and multivariable logistic regression analyses identified DM, impaired RV-PA coupling, lower LVEF, smaller LV end-diastolic dimension, and larger LA dimension before M-TEER as factors associated with impaired RV-PA coupling (Table 5).

**Table 5:** Univariable and Multivariable Logistic Regression Analyses for Predicting the Impaired RV-PA Coupling After M-TEER

	Univariable Model		Multivariable model		VIF
	OR (95% CI)	*P* Value	OR (95% CI)	*P* Value	
Age, y	1.020 (1.000–1.030)	0.021	1.010 (0.988-1.040)	0.292	1.36
Male	0.697 (0.532-0.913)	0.009	0.923 (0.574-1.490)	0.742	1.34
BMI, kg/m^2^	0.983 (0.947–1.020)	0.382	0.997 (0.937-1.060)	0.937	1.23
Clinical frailty scale	1.090 (0.966-1.230)	0.159	1.050 (0.857-1.280)	0.645	1.20
AF/AFL	1.460 (1.110-1.920)	0.007	1.230 (0.778-1.950)	0.376	1.23
Hypertension	1.060 (0.808-1.390)	0.671	0.867 (0.563-1.340)	0.518	1.13
Diabetes mellitus	1.240 (0.941-1.620)	0.128	1.720 (1.120-2.660)	0.014	1.13
Hospitalization for HF	0.956 (0.638-1.430)	0.829	0.756 (0.408-1.400)	0.373	1.09
Previous cardiac surgery	2.120 (1.440-3.130)	<0.001	1.460 (0.815-2.630)	0.202	1.12
Coronary artery disease	1.150 (0.884-1.500)	0.297	0.952 (0.603-1.500)	0.834	1.33
CRT	1.140 (0.803-1.630)	0.458	1.090 (0.610-1.950)	0.771	1.20
Hb, g/dL	0.920 (0.858-0.988)	0.021	0.943 (0.839-1.060)	0.319	1.25
eGFR, 10 mL/min/1.73 m^2^	0.895 (0.832-0.963)	0.003	1.010 (0.890-1.140)	0.905	1.26
Log BNP, pg/mL	2.450 (1.690-3.550)	<0.001	1.610 (0.911-2.840)	0.102	1.46
Systolic blood pressure, mm Hg	0.996 (0.988-1.000)	0.258	0.987 (0.974-1.000)	0.064	1.23
LVEF, 5%	1.000 (1.000-1.000)	0.982	0.843 (0.738-0.962)	0.011	1.79
LVDd, mm	0.982 (0.968-0.996)	0.014	0.954 (0.924-0.984)	0.003	2.03
LAD, mm	1.030 (1.010-1.040)	<0.001	1.040 (1.010-1.070)	0.011	1.65
ERO, cm^2^	1.750 (0.773-3.970)	0.179	0.583 (0.151-2.250)	0.434	1.27
TR ≥ moderate to severe	1.680 (1.070-2.660)	0.025	1.700 (0.813-3.560)	0.158	1.12
Impaired RV-PA coupling before M-TEER	4.980 (3.750-6.600)	<0.001	4.510 (2.940-6.900)	<0.001	1.12
MR after M-TEER ≥ moderate	1.190 (0.861-1.650)	0.288	0.852 (0.497-1.460)	0.558	1.13
TMG after M-TEER ≥4 mm Hg	1.130 (1.030-1.240)	0.011	1.420 (0.838-2.390)	0.194	1.11

VIF = variance inflation factor; other abbreviations as in Table 1, Table 2, Table 3.

## Discussion

In this study, we evaluated the prognostic significance and clinical predictors of impaired RV-PA coupling after M-TEER in patients with ventricular FMR. Our findings indicated that: 1) impaired RV-PA coupling after M-TEER was independently linked to an increased risk of cardiovascular events in patients with ventricular FMR; 2) RV-PA coupling after M-TEER is effective for stratifying prognosis in patients with PH after M-TEER; and 3) DM, impaired RV-PA coupling before M-TEER, and smaller LV size at baseline are associated with impaired RV-PA coupling.

### Postprocedural RV-PA coupling and outcomes in ventricular FMR after M-TEER

Our findings suggest that RV-PA coupling after M-TEER is a strong prognostic indicator in patients with ventricular FMR. TAPSE alone was not predictive of the outcome. Although previous studies have reported the utility of preprocedural RV-PA coupling and its periprocedural changes in patients with ventricular FMR,^12^, ^13^, ^14^^,^^24^^,^^25^ preprocedural RV-PA coupling was not significantly associated with clinical outcomes. Postprocedural RV-PA coupling likely reflects a combination of pre-existing RV–pulmonary vascular dysfunction and dynamic hemodynamic changes following M-TEER. Therefore, it should be interpreted as a prognostic marker rather than a mechanistic determinant of outcome.

Trajectory analysis of TAPSE and SPAP showed that patients with impaired RV-PA coupling had limited or even reduced TAPSE after M-TEER compared with those with preserved RV-PA coupling. Although SPAP generally improved after the procedure, the improvement was significantly smaller in patients with impaired RV-PA coupling, compared with those with preserved RV-PA coupling, and SPAP remained elevated in this group.

A previous study showed that M-TEER reduces SPAP in patients with PH,^26^ whereas SPAP may paradoxically increase in those without PH. Contributing factors include iatrogenic aortic septal defect, elevated mitral valve pressure gradient, and impaired LA compliance.^27^, ^28^, ^29^, ^30^ Although iatrogenic aortic septal defect can relieve LA pressure and reduce pulmonary capillary wedge pressure,^30^ the resultant left-to-right shunt may lead to RV dilatation and elevated pulmonary pressures.^31^

### RV-PA coupling in patients with and without PH

Guazzi et al^32^ previously reported that impaired RV-PA coupling in PH is associated with reduced functional capacity and worse clinical outcomes. Our findings extend this by demonstrating that RV-PA coupling provides effective prognostic stratification, specifically in patients with PH.

In contrast, its utility in patients without PH is limited, which may be explained by 2 factors. First, the prognostic impact of PH may overshadow the contribution of RV-PA coupling. Accordingly, previous studies have shown that elevated SPAP levels are associated with worse clinical outcomes in patients with HF and severe ventricular FMR undergoing M-TEER.^33^^,^^34^ Second, TAPSE and SPAP may have been overestimated or underestimated. Although TAPSE is a reproducible and widely used marker of RV function, it reflects only RV basal longitudinal motion and is sensitive to the image angle and loading conditions.^20^ Similarly, SPAP estimation via the peak tricuspid regurgitant velocity using the simplified Bernoulli equation is prone to underestimation, particularly in cases with severe or suboptimal TR signals.

### Predictors of impaired RV-PA coupling

This study identified DM, impaired RV-PA coupling, lower LVEF, smaller LV size, and larger LA size before M-TEER as independent predictors of impaired RV-PA coupling after M-TEER. DM has been linked to subclinical RV dysfunction via myocardial fibrosis and microvascular disease.^35^ Although the correlation between LV size and RV size has not been assessed, the potential impact of pericardial interaction resulting from RV volume and pressure overload should be considered.^36^

### Study limitations

First, a substantial proportion of patients had missing TAPSE and/or SPAP data, which may have introduced selection bias. Although IPW with balance diagnostics and ESS assessment was performed as a sensitivity analysis, residual bias due to unmeasured predictors of missingness or outcome, or violations of the missing at random assumption, cannot be entirely excluded. Although center-level variation theoretically could be considered for instrumental variable analysis, the present multicenter cohort did not provide a sufficiently strong or valid instrument, and such analyses were therefore not performed. Second, the echocardiographic assessment was not reviewed by a central core laboratory, although it was performed by experienced echocardiographers at each hospital. Third, right atrial pressure was standardized at 8 mm Hg in accordance with ASE recommendations.^20^ Because this multicenter retrospective study included institutions with heterogeneous right atrial pressure estimation methods, standardization was performed to reduce intercenter variability and ensure consistency in SPAP calculation. Although this approach may have resulted in over- or underestimation of SPAP, it provided a uniform basis for group comparisons. Furthermore, the prevalence of severe TR was low (0.7%) and did not differ between groups (*P* = 1.000), suggesting limited impact on the overall precision of RV-PA coupling assessment. Fourth, TAPSE reflects the longitudinal displacement of a single RV segment and is angle-dependent, making echocardiographic RV-PA coupling inherently technique-dependent.^20^ In addition, TR velocity–based SPAP estimation may be less reliable in the presence of severe TR. Cardiac magnetic resonance imaging provides more reproducible and comprehensive RV phenotyping, particularly in patients with PH.^37^ However, cardiac magnetic resonance imaging data were not available in the present study. Fifth, given the significant differences in baseline characteristics between the impaired and normal RV-PA coupling groups, causal relationships cannot be definitively established. Although multivariable adjustment was performed, residual confounding due to unmeasured or incompletely measured variables may have influenced the observed associations. Specifically, BNP and/or NT-proBNP data were unavailable for a substantial proportion of patients because of the retrospective nature of the study. Although we performed supplementary analyses incorporating these biomarkers, the large amount of missing data resulted in reduced statistical power and potential selection bias. Accordingly, although our findings support the independent prognostic value of RV-PA coupling, the influence of residual confounding related to incomplete baseline variables cannot be fully excluded. Sixth, because of the retrospective design of this study conducted between 2018 and 2023, the study population reflects a period preceding the widespread adoption of contemporary HF therapies. Specifically, the low prescription rates of sodium-glucose cotransporter 2 inhibitors and angiotensin receptor neprilysin inhibitors may limit the generalizability of our findings to patients receiving current guideline-directed medical therapy. Seventh, subgroup and interaction analyses should be interpreted with caution. The relatively small sample size within certain subgroups may have resulted in unstable HR estimates and limited statistical power to detect significant interaction effects. Finally, changes in RV-PA coupling during the chronic phase after M-TEER were not evaluated.

## Conclusions

Impaired RV-PA coupling after M-TEER is independently associated with a higher risk of composite outcomes in patients with ventricular FMR. Our findings demonstrate that RV-PA coupling remained a significant prognostic factor even within the subgroup of patients with PH after M-TEER. This underscores the clinical utility of RV-PA coupling by providing incremental prognostic information beyond conventional markers, particularly for identifying high-risk individuals with persistent PH.

## Funding Support and Author Disclosures

The OCEAN-Mitral registry is part of the OCEAN-SHD registry and is supported by Edwards Lifesciences, Medtronic Japan, Boston Scientific, Abbott Medical Japan, and Daiichi-Sankyo Company. Drs Yamamoto, Asami, Izumi, Saji, Enta, Shirai, Izumo, Kuwata, Mizuno, Watanabe, Amaki, Bota, Ohno, Ueno, Kubo, and Hayashida are clinical proctors of M-TEER for Abbott Medical. Drs Otsuki, Yamamoto, Asami, Enta, Saji, Shirai, Izumo, Kuwata, Mizuno, Watanabe, Amaki, Kodama, Bota, Ohno, and Kubo have received lecturer fees from Abbott Medical. Drs Yamamoto, Saji, Enta, Izumo, Kuwata, Shirai, Mizuno, Watanabe, Amaki, Bota, Ohno, Kubo, and Ohno have received consultant fees from Abbott Medical. Dr Otsuki has received a scholarship donation from Abbott Medical. Dr Ohno is an advisor for Abbott Medical. All other authors have reported that they have no relationships relevant to the contents of this paper to disclose.
