# Supplementary material for: Right Ventricular–to–Pulmonary Artery Uncoupling Following Transcatheter Edge-to-Edge Repair in Ventricular Functional Mitral Regurgitation
Source: JACC Asia. 2026 Jun 5;6(9):1931–43. doi: 10.1016/j.jacasi.2026.03.040 (PMC13554777; doi:10.1016/j.jacasi.2026.03.040)
Supplement: Supplementary file 1 [file mmc1.docx]

**Supplemental Figure 1.** Kaplan−Meier survival curve for a composite endpoint in the sensitivity analysis (p = 0.524).

**Supplemental Figure 2** Kaplan−Meier survival curve for a composite endpoint in the sensitivity analysis (p < 0.001).

M-TEER: Mitral transcatheter edge-to-edge repair; RV-PA: right ventricular–pulmonary artery.

**Supplemental Figure 3.**

Kaplan−Meier survival curves between impaired and preserved RV-PA coupling before M-TEER

(A) Composite endpoints (cardiovascular death and hospitalization for heart failure) (p = 0.264), (B) cardiovascular death (p = 0.408) and (C) hospitalization for heart failure (p = 0.477).

M-TEER: Mitral transcatheter edge-to-edge repair; RV-PA: right ventricular–pulmonary artery.

**Supplemental Figure 4.** Kaplan−Meier survival curves stratified by RV-PA coupling before and after M-TEER

M-TEER: Mitral transcatheter edge-to-edge repair; RV-PA: right ventricular–pulmonary artery.

**Supplemental Table 1.** Missing data

|  | Missing data  (n=584) | This cohort  (n=1,059) | p-value |
| --- | --- | --- | --- |
| Sex, male | 366 (62.7) | 687 (64.9) | 0.403 |
| Age, years | 77.0 (71.0-83.0) | 77.0 (70.0-82.0) | 0.478 |
| BMI, kg/m^2^ | 21.1 (18.7-23.5) | 21.4 (19.1-23.5) | 0.321 |
| BSA, m^2^ | 1.54 (1.40-1.68) | 1.55 (1.41-1.67) | 0.516 |
| Hypertension | 378 (64.7) | 671 (63.4) | 0.619 |
| Diabetes mellitus | 215 (36.8) | 359 (33.9) | 0.258 |
| CKD | 512 (87.7) | 942 (89.0) | 0.485 |
| Dialysis | 51 (8.7) | 86 (8.1) | 0.737 |
| Dyslipidemia | 330 (56.5) | 583 (55.1) | 0.606 |
| Coronary artery disease | 295 (50.5) | 457 (43.2) | 0.329 |
| COPD | 58 (9.9) | 91 (8.3) | 0.415 |
| AF/AFL | 310 (53.1) | 642 (60.6) | 0.004 |
| Previous cardiac surgery | 55 (9.4) | 117 (11.0) | 0.343 |
| NYHA Ⅲ-Ⅳ | 352 (60.3) | 634 (59.9) | 0.914 |
| Clinical frailty scale | 4.0 (3.0-4.0) | 3.0 (3.0-4.0) | 0.002 |
| β-blocker | 497 (85.1) | 916 (86.5) | 0.481 |
| ACE inhibitor/ARB | 298 (51.0) | 627 (59.5) | 0.001 |
| ARNI | 104 (17.8) | 112 (10.6) | <0.001 |
| MRA | 343 (58.7) | 632 (59.7)) | 0.748 |
| Diuretics | 482 (82.5) | 934 (88.2) | 0.002 |
| SGLT2 inhibitor | 191 (32.7) | 257 (24.3) | <0.001 |
| EuroSCORE Ⅱ | 5.23 (3.50-8.45) | 5.64 (3.48-8.83) | 0.390 |
| STS | 8.92 (5.08-13.68) | 8.70 (5.32-13.56) | 0.948 |
| Hb, g/dL | 11.7 (10.4-13.2) | 12.0 (10.8-13.3) | 0.028 |
| eGFR, ml/min/1.73m^2^ | 37.3 (24.4-48.9) | 35.9 (23.8-49.0) | 0.546 |
| BNP, pg/mL | 485.8 (218.1-948.3) | 416.6 (231.8-768.8) | 0.122 |
| NT-pro BNP, pg/mL | 3238.0 (1671.6-6463.6) | 3235.8 (1622.8-6062.5) | 0.923 |

Median (IQR), or n (%).

ACE: angiotensin-converting enzyme; AF: atrial fibrillation; AFL: atrial flutter; ARB: angiotensin Ⅱ receptor blocker; ARNI: angiotensin receptor-neprilysin inhibitor; BMI: body mass index; BNP: B-type natriuretic peptide; BSA: body surface area; COPD: chronic obstructive pulmonary disease; CKD: chronic kidney disease; Cre: creatinine; CRT: cardiac resynchronization therapy; eGFR: estimated glomerular filtration rate; EuroScore: European system of cardiac operative risk evaluation; Hb: hemoglobin; MRA: mineralocorticoid receptor antagonist; NYHA: New York Heart Association functional class; NT-pro BNP: N-terminal pro-B type natriuretic peptide; SGLT: sodium-glucose cotransporter 2; STS: society of thoracic surgeons risk score.

**Supplemental Table 2 Baseline characteristics of patients with and without missing TAPSE and SPAP data and standardized mean differences**

|  | Missing data  (n=472) | This cohort  (n=894) | p-value | SMD |
| --- | --- | --- | --- | --- |
| Sex, male | 292 (61.9) | 591 (66.1) | 0.134 | 0.088 |
| Age, years | 78.0 (72.0-83.0) | 77.0 (70.0-82.0) | 0.176 | 0.058 |
| BMI, kg/m^2^ | 21.1 (18.7-23.5) | 21.4 (19.1-23.4) | 0.266 | 0.047 |
| Diabetes mellitus | 170 (36.0) | 299 (33.4) | 0.372 | 0.054 |
| AF/AFL | 250 (53.0) | 545 (61.0) | 0.005 | 0.162 |
| NYHA Ⅲ-Ⅳ | 292 (61.9) | 545 (61.0) | 0.789 | 0.019 |
| Clinical frailty scale | 4.0 (3.0-4.0) | 3.0 (3.0-4.0) | 0.003 | 0.178 |
| eGFR, ml/min/1.73m^2^ | 36.9 (23.3-48.8) | 37.0 (24. -49.2) | 0.609 | 0.032 |
| After M-TEER |  |  |  |  |
| Systolic blood pressure, mmHg | 106.0 (95.0-120.0) | 104.0 (94.0-117.0) | 0.031 | 0.154 |
| LVEF, % | 31.5 (25.3-38.1) | 31.3 (25.0-39.0) | 0.789 | 0.026 |
| MR ≥ moderate | 74 (15.7) | 161 (18.0) | 0.312 | 0.062 |
| TR ≥ moderate to severe | 13 (2.8) | 46 (5.1) | 0.054 | 0.123 |
| TMG ≥ 4mmhg | 94 (19.9) | 172 (19.2) | 0.820 | 0.017 |

AF: atrial fibrillation; AFL: atrial flutter; BMI: body mass index; eGFR: estimated glomerular filtration rate; LVEF: left ventricular ejection fraction; MR: mitral regurgitation; New York Heart Association functional classification; SMD: standardized mean differences; TMG: trans mitral gradient and TR: tricuspid regurgitation.

**Supplemental Table 3. Baseline characteristics**

|  | Impaired RV-PA coupling before M-TEER and  impaired RV-PA coupling after-M-TEER  (n=223) | Normal RV-PA coupling before M-TEER and  impaired RV-PA coupling after-M-TEER  (n = 102) | Impaired RV-PA coupling before M-TEER and  normal RV-PA coupling after-M-TEER  (n = 224) | Normal RV-PA coupling before M-TEER and  normal RV-PA coupling after-M-TEER  (n=510) | p-value |
| --- | --- | --- | --- | --- | --- |
| Sex, male | 132 (59.2) | 60 (58.8) | 155 (69.2) | 340 (66.7) | 0.061 |
| Age, years | 77.0 (71.0–82.0) | 80.0 (74.0– 84.0) | 76.0 (70.0– 82.0) | 77.0 (70.0–82.8) | 0.016 |
| BMI, kg/m^2^ | 21.3 (19.1– 22.8) | 21.5 (19.2– 23.2) | 21.4 (19.0– 23.3) | 21.3 (19.0– 23.7) | 0.806 |
| BSA, m^2^ | 1.51 (1.38– 1.64) | 1.56 (1.38–1.69) | 1.56 (1.42–1.67) | 1.56 (1.43– 1.68) | 0.035 |
| Hypertension | 138 (61.9) | 71 (69.6) | 153 (68.3) | 309 (60.6) | 0.113 |
| Diabetes mellitus | 90 (40.4) | 31 (30.4) | 85 (37.9) | 153 (30.0) | 0.02 |
| CKD | 204 (91.5) | 93 (91.2) | 200 (89.3) | 445 (87.3) | 0.323 |
| Dialysis | 27 (12.1) | 4 (3.9) | 19 (8.5) | 36 (7.1) | 0.047 |
| Dyslipidemia | 120 (53.8) | 52 (51.0) | 125 (55.8) | 286 (56.1) | 0.779 |
| Coronary artery disease | 107 (48.0) | 41 (40.2) | 106 (47.3) | 203 (39.8) | 0.094 |
| Previous PCI | 83 (37.2) | 32 (31.4) | 86 (38.4) | 174 (34.1) | 0.514 |
| Previous CABG | 35 (15.7) | 7 (6.9) | 16 (7.1) | 17 (3.3) | <0.001 |
| COPD | 20 (9.0) | 10 (9.8) | 24 (10.7) | 37 (7.3) | 0.444 |
| AF/AFL | 147 (65.9) | 70 (68.6) | 125 (55.8) | 300 (58.8) | 0.042 |
| Prior hospitalization for Heart failure | 191 (87.6) | 85 (87.6) | 192 (87.7) | 437 (88.3) | 0.992 |
| Previous cardiac surgery | 44 (19.7) | 10 (9.8) | 29 (12.9) | 34 (6.7) | <0.001 |
| CRT | 42 (18.8) | 13 (12.7) | 32 (14.3) | 79 (15.5) | 0.443 |
| NYHA Ⅲ-Ⅳ | 160 (71.7) | 58 (56.9) | 145 (64.7) | 271 (53.1) | <0.001 |
| Clinical frailty scale | 3.00 (3.00–4.00) | 3.00 (3.00–4.00) | 3.00 (3.00–4.00) | 3.00 (3.00–4.00) | 0.447 |
| β-blocker | 192 (86.1) | 88 (86.3) | 200 (89.3) | 436 (85.5) | 0.579 |
| ACE inhibitor/ARB | 126 (56.5) | 60 (58.8) | 133 (59.4) | 308 (60.4) | 0.806 |
| ARNI | 16 (7.2) | 14 (13.7) | 26 (11.6) | 56 (11.0) | 0.247 |
| MRA | 125 (56.1) | 61 (59.8) | 147 (65.6) | 299 (58.6) | 0.192 |
| Diuretics | 196 (87.9) | 93 (91.2) | 205 (91.5) | 440 (86.3) | 0.167 |
| SGLT2 inhibitor | 54 (24.2) | 25 (24.5) | 58 (25.9) | 120 (23.5) | 0.924 |
| EuroSCORE Ⅱ | 6.60 (4.03–11.78) | 5.34 (3.92– 10.44) | 5.59 (3.80–8.80) | 4.90 (3.04– 7.99) | <0.001 |
| STS | 9.98 (6.30– 16.33) | 8.84 (5.93– 13.13) | 8.98 (5.30–13.55) | 7.99 (4.76–12.27) | <0.001 |
| Hb, g/dL | 11.7 (10.5– 12.9) | 11.9 (10.8–13.4) | 12.1 (10.9–13.5) | 12.1 (10.8– 13.4) | 0.078 |
| Cre, mg/dL | 1.50 (1.13–2.17) | 1.29 (0.99–1.85) | 1.32 (1.05–2.04) | 1.31 (1.00–1.80) | 0.004 |
| eGFR, ml/min/1.73 m^2^ | 32.2 (21.5– 42.3) | 38.1 (24.1– 49.3) | 36.9 (24.3–48.8) | 37.3 (25.9–51.0) | 0.001 |
| BNP, pg/mL | 621.1 (350.7– 1209.1) | 352.8 (196.0– 729.4) | 500.6 (257.7– 937.6) | 339.3 (165.0– 568.9) | <0.001 |
| NT-pro BNP, pg/mL | 5270.5 (3280.5– 11412.3) | 2353.5 (1459.0– 5176.8) | 3568.0 (1800.5– 6608.5) | 2528.0 (1398.0–4531.5) | <0.001 |
| Number of clips  1  2  3 | 156 (70.0)  65 (29.1)  2 (0.9) | 69 (67.6)  30 (29.4)  3 (2.9) | 163 (72.8)  61 (27.2)  0 (0) | 380 (74.5)  124 (24.3)  6 (1.2) | 0.190 |

Data are presented as median (IQR) or n (%).

ACE: angiotensin-converting enzyme; AF: atrial fibrillation; AFL: atrial flutter; ARB: angiotensin Ⅱ receptor blocker; ARNI: angiotensin receptor-neprilysin inhibitor; BMI: body mass index; BNP: B-type natriuretic peptide; BSA: body surface area; CABG: coronary artery bypass grafting; COPD: chronic obstructive pulmonary disease; CKD: chronic kidney disease; Cre: creatinine; CRT: cardiac resynchronization therapy; eGFR: estimated glomerular filtration rate; EuroScore: European system of cardiac operative risk evaluation; Hb: hemoglobin; MRA: mineralocorticoid receptor antagonist; NYHA: New York Heart Association functional class; NT-pro BNP: N-terminal pro-B type natriuretic peptide; PCI: percutaneous coronary artery intervention; SGLT: sodium-glucose cotransporter 2; STS: society of thoracic surgeons risk score.

**Supplemental Table 4** **Echocardiographic characteristics before M-TEER**

|  | Impaired RV-PA coupling before M-TEER and  impaired RV-PA coupling after-M-TEER  (n=223) | Normal RV-PA coupling before M-TEER and  impaired RV-PA coupling after-M-TEER  (n = 102) | Impaired RV-PA coupling before M-TEER and  normal RV-PA coupling after-M-TEER  (n = 224) | Normal RV-PA coupling before M-TEER and  normal RV-PA coupling after-M-TEER  (n=510) | p-value |
| --- | --- | --- | --- | --- | --- |
| Systolic blood pressure, mmHg | 102.0 (94.0–114.0) | 102.5 (95.3–117.0) | 104.0 (92.0–116.0) | 106.0 (95.3–120.0) | 0.163 |
| Heart rate, beats/min | 74.0 (67.5–84.5) | 73.0 (65.0–82.8) | 71.0 (63.0–80.0) | 70.0 (61.0–80.0) | 0.005 |
| LVDd, mm | 61.0 (55.0–67.0) | 59.0 (53.0–66.8) | 63.0 (58.0–69.0) | 61.0 (55.0–67.0) | 0.005 |
| LVDs, mm | 51.0 (43.0–58.0) | 49.0 (40.0–57.0) | 54.0 (47.0–60.3) | 51.0 (44.4–58.0) | 0.002 |
| LVEDV, mL | 171.0 (126.0–208.0) | 152.0 (105.0–201.0) | 181.0 (141.8–224.3) | 165.0 (125.0–210.5) | 0.007 |
| LVESV, mL | 110.0 (72.5–151.0) | 96.5 (62.5–139.3) | 124.0 (84.5–160.0) | 108.0 (74.0–147.0) | 0.007 |
| LVEF, % | 32.7 [27.2–40.8] | 35.1 [27.9–42.6] | 31.2 (26.3–38.4) | 33.4 (27.2–41.0) | 0.147 |
| LAD, mm | 51.0 (46.0–56.0) | 49.0 (45.0–56.0) | 50.0 (45.0–54.5) | 47.0 (43.0–53.0) | <0.001 |
| LAV index, mL/m^2^ | 81.2 (64.2–110.5) | 82.3 (64.9–105.6) | 73.8 (59.3–98.4) | 67.1 (52.7–90.3) | <0.001 |
| Stroke volume, mL | 40.9 (32.8–52.1) | 43.4 (37.9–55.6) | 43.8 [33.1–57.0] | 48.0 (37.4–60.7) | <0.001 |
| SPAP, mmHg | 49.0 (40.0–59.0 | 34.0 (30.2–40.0) | 49.0 (40.0–60.0) | 33.0 (28.0–38.8) | <0.001 |
| TAPSE, mm | 12.4 (10.4–14.2) | 16.0 (13.5–18.8) | 13.3 (11.0–15.6) | 17.8 (15.0–20.3) | <0.001 |
| TAPSE/SPAP | 0.25 (0.21– 0.30) | 0.45 (0.39–0.54) | 0.28 (0.23–0.32) | 0.51 (0.43–0.62) | <0.001 |
| RV s’, cm/s | 7.6 (6.1–9.4) | 8.8 (6.9–9.8) | 8.0 (7.0–9.9) | 9.3 (7.7–11.2) | <0.001 |
| FAC, % | 29.6 (24.0–37.0) | 34.0 (28.0–39.0) | 31.0 (23.0–37.0) | 39.0 (31.0–46.0) | <0.001 |
| RVDd-mid, mm | 30.9 (26.5–35.9] | 31.1 (27.8–35.3) | 31.1 (25.4–35.2] | 28.1 (24.9–33.3) | 0.001 |
| MR grade  ≤ moderate  Moderate to severe  Severe | 19 (8.5)  75 (33.6)  129 (57.8) | 18 (17.6)  42 (41.2)  42 (41.2) | 27 (12.0)  64 (28.6)  133 (59.4) | 98 (19.2)  167 (32.7)  245 (48.0) | 0.004 |
| MR ERO, cm^2^ | 0.33 (0.26–0.44) | 0.26 (0.21–0.33) | 0.32 (0.25–0.41) | 0.30 (0.21–0.39) | <0.001 |
| TR grade  Moderate  Moderate to severe  Severe | 80 (35.9)  19 (8.5)  3 (1.3) | 28 (27.5)  11 (10.8)  2 (2.0) | 83 (37.1)  12 (5.4)  1 (0.4) | 92 (18.1)  32 (6.3)  4 (0.8) | < 0.001 |
| E/e’ | 19.4 (15.1–24.3) | 15.7 (11.3–19.9) | 17.8 (14.3–23.9) | 15.1 (11.3–19.4) | <0.001 |
| E, cm/s | 105.0 (89.0–122.0) | 88.0 (68.0–106.0) | 99.0 (84.0–117.3) | 84.2 (65.8–104.0] | <0.001 |

Data are presented as median (IQR) or n (%).

ERO: effective regurgitant orifice area; FAC: fractional area change ;LAD: left atrial dimension; LAV: left atrial volume; LVDd: left ventricular diastolic dimension; LVDs: left ventricular systolic dimension; LVEDV: left ventricular end-diastolic volume; LVESV: left ventricular end-systolic volume; LVEF: left ventricular ejection fraction; MR: mitral regurgitation; RV: right ventricular; RVDd: right ventricular diastolic dimension; SPAP: systolic pulmonary artery pressure; TAPSE: tricuspid annular plane systolic excursion; TR: tricuspid regurgitation.

**Supplemental Table 5 Echocardiographic characteristics after M-TEER**

|  | Impaired RV-PA coupling before M-TEER and  impaired RV-PA coupling after-M-TEER  (n=223) | Normal RV-PA coupling before M-TEER and  impaired RV-PA coupling after-M-TEER  (n = 102) | Impaired RV-PA coupling before M-TEER and  normal RV-PA coupling after-M-TEER  (n = 224) | Normal RV-PA coupling before M-TEER and  normal RV-PA coupling after-M-TEER  (n=510) | p-value |
| --- | --- | --- | --- | --- | --- |
| Systolic blood pressure, mmHg | 105.0 (95.0–114.0) | 102.0 (95.0–117.0) | 102.0 (92.0–114.0) | 102.0 (92.0–116.0) | 0.485 |
| Heart rate, beats/min | 70.0 (63.0–79.0) | 70.0 (64.0–80.0) | 69.5 (61.0–79.0) | 68.0 (60.0–75.0) | 0.015 |
| LVDd, mm | 60.0 (52.0–65.6) | 56.5 (50.0–65.0) | 61.0 (55.0–67.0) | 60.0 (54.0–66.0) | 0.004 |
| LVDs, mm | 50.5 (41.7–59.0) | 46.0 (39.0–57.0) | 54.0 (46.0–60.0) | 51.0 (43.0–58.0) | 0.003 |
| LVEDV, mL | 161.0 (115.0–211.0) | 141.0 (107.0–196.0) | 169.5 (133.0–213.5) | 155.0 (117.3–199.8) | 0.022 |
| LVESV, mL | 109.0 (72.5– 153.5) | 93.0 (63.0–144.5) | 120.0 (82.5–156.8) | 103.0 (74.0–141.0) | 0.017 |
| LVEF, % | 28.9 (24.2–37.5) | 33.1 (27.5–40.9) | 30.2 (24.4–37.23) | 32.2 (25.7–40.4) | 0.025 |
| LAD, mm | 49.9 (45.0–54.0) | 48.0 (43.0–55.0) | 48.0 (43.0–53.0) | 46.0 (41.0–52.0) | <0.001 |
| LAV index, mL/m^2^ | 78.2 (59.8– 103.4) | 75.8 (61.8–102.6) | 67.2 (54.9–88.5) | 65.3 (50.5–87.1) | <0.001 |
| Stroke volume, mL | 41.5 (34.4–53.1) | 44.9 (34.3–54.6) | 48.0 (38.0–62.7) | 49.1 (39.9–60.8) | <0.001 |
| SPAP, mmHg | 44.0 (37.0–52.0) | 37.5 (34.0–45.0) | 34.9 (30.0–41.0) | 32.0 (28.0–37.0) | <0.001 |
| ΔSPAP, mmHg | -5.0 (-12.0– 1.0) | 4.0 (-0.8–10.1) | -14.7 (-24.0–4.8) | -1.0 (-5.8–4.0) | <0.001 |
| TAPSE, mm | 12.0 (10.0–14.0) | 11.7 (9.5–13.7) | 16.6 (13.9–19.0) | 17.4 (15.0–20.1) | <0.001 |
| ΔTAPSE | 0.0 (-2.2–1.5) | -4.2 (-7.4–-2.0) | 3.0 (0.5–5.2) | 0.0 (-2.8–2.2) | <0.001 |
| TAPSE/SPAP | 0.28 (0.24–0.32) | 0.31 (0.26–0.34) | 0.45 (0.40–0.53) | 0.52 (0.45–0.62) | <0.001 |
| ΔTAPSE/SPAP | 0.02 (-0.02–0.07) | -0.17 (-0.24– -0.1) | 0.19 (0.12–0.26) | 0.01 (-0.08–0.09) | <0.001 |
| MR grade  Moderate | 47 (21.1) | 21 (20.6) | 40 (17.9) | 93 (18.2) | 0.969 |
| TMG, mmHg | 2.90 (2.0–3.62) | 2.50 (1.90–3.8) | 2.30 (1.80–3.10) | 2.40 (1.90–3.38) | 0.037 |
| TR, grade  Moderate  Moderate to severe  Severe | 57 (25.6)  17 (7.6)  1 (0.4) | 28 (27.5)  10 (9.8)  1 (1.0) | 47 (21.0)  3 (1.3)  2 (0.9) | 70 (13.8)  27 (5.3)  3 (0.6) | < 0.001 |
| E/e’ | 25.2 (20.5–32.6) | 24.4 (18.5–30.9) | 24.8 (20.7–31.1) | 22.0 (17.5–29.1) | 0.003 |
| E, cm/s | 114.0 (90.7–138.0) | 106.4 (70.0–136.0) | 105.0 (84.5–125.5) | 95.0 (72.0–116.0) | <0.001 |

Data are presented as median (IQR) or n (%).

LAD: left atrial dimension; LAV: left atrial volume; LVDd: left ventricular diastolic dimension; LVDs: left ventricular systolic dimension; LVEDV: left ventricular end-diastolic volume; LVESV: left ventricular end-systolic volume; LVEF: left ventricular ejection fraction; MR: mitral regurgitation; SPAP: systolic pulmonary artery pressure; TAPSE: tricuspid annular plane systolic excursion; TMG: trans mitral gradient; TR: tricuspid regurgitation.

Supplemental Table 6 Variance inflation factors of covariates

|  | VIF |
| --- | --- |
| Variables |  |
| Age, year | 1.15 |
| Male | 1.14 |
| BMI, kg/m^2^ | 1.11 |
| Clinical frailty scale | 1.14 |
| AF/AFL | 1.14 |
| Diabetes mellitus | 1.06 |
| NYHA Ⅲ-Ⅳ | 1.10 |
| eGFR before M-TEER, ml/min/1.73m^2^ | 1.06 |
| Systolic blood pressure after M-TEER, mmHg | 1.19 |
| LVEF after M-TEER, % | 1.25 |
| TR after M-TEER ≥ 3+ | 1.04 |
| Impaired RV-PA coupling after M-TEER | 1.03 |
| MR after M-TEER ≥ 2+ | 1.04 |
| TMG after M-TEER ≥ 4mmhg | 1.10 |

AF: atrial fibrillation; AFL: atrial flutter; BMI: body mass index; BNP: B-type natriuretic peptide; eGFR: estimated glomerular filtration rate; LVEF: left ventricular ejection fraction; MR: mitral regurgitation; M-TEER: Mitral transcatheter edge-to-edge repair; TMG: trans mitral gradient; TR: tricuspid regurgitation; VIF: variance inflation factor.
